# Long‐term outcomes following the resection of screen‐detected right‐sided colon cancer

**DOI:** 10.1002/wjs.12409

**Published:** 2024-11-19

**Authors:** James Lucocq, Thomas Trinder, Elli Symeonidou, Katy Homyer, Hassan Baig, Pradeep Patil, Girivasan Muthukumarasamy

**Affiliations:** ^1^ Ninewells Hospital Dundee UK; ^2^ University Hospital Ayr UK; ^3^ Western General Hospital Edinburgh UK; ^4^ Victoria Hospital Kirkcaldy Kirkcaldy UK

**Keywords:** colon cancer, recurrence, resection, screening, survival

## Abstract

**Background:**

The relative outcomes following the resection of screen‐detected right‐sided colon cancer compared to symptomatic cases are unknown. In this study, short and long‐term outcomes after right‐sided colectomy in screen‐detected colon cancer are compared with symptomatic cases, both emergency and elective.

**Methods:**

A prospective observational cohort study of patients, including both screen‐detected and symptomatic patients (elective and emergency resections), undergoing right‐sided colectomy for colon cancer (2010–2020) in a tertiary care unit was conducted. Each patient was followed up for long‐term recurrence and survival.

**Results:**

A total of 909 patients (median age, 70; IQR, 58–82; male, 52%) were included (151 patients (16.6%) screen‐detected; 598 (65.8%) elective and 160 (17.6%) emergency). Screen‐detected patients were more likely to have T1 or T2 lesions compared to elective and emergency groups (T1: 14.6% vs. 3.8% vs. 0.6% *p* < 0.001; T2: 16.6% vs. 8.9% vs. 3.1% *p* < 0.001), but were less likely to have T3 or T4 lesions (*p* < 0.001), respectively. Rates of N0 were higher in the screen‐detected group (68.9% vs. 63.5% vs. 41.9%, respectively; *p* < 0.001). 98% of the screen‐detected group achieved R0 resection compared to 93.3% of elective and 79.4% of emergency patients (*p* < 0.001). At 5‐years following resection, overall survival for the screen‐detected, elective, and emergency groups were 85.4%, 75.4%, and 53.1%, respectively (*p* < 0.001). Recurrence at 5‐year post‐resection were 8%, 15.1%, and 22.5% for the screen‐detected, elective, and emergency groups, respectively (*p* < 0.001).

**Discussion:**

When considering right‐sided colon cancer alone, screen‐detected cancers have a lower long‐term recurrence rate, lower rates of postoperative complication, and superior survival compared to symptomatic groups following resection.

## 1 INTRODUCTION

Colorectal cancer is one of the most common cancer diagnoses worldwide, with high rates of morbidity and mortality.^1^, ^2^, ^3^ While patient outcomes have benefitted from the introduction of screening programs since 2006 through the early identification of asymptomatic cancers, peri‐operative outcomes compared with symptomatic patients undergoing either elective (non‐screen detected) or emergency colectomy are still largely unknown. While some studies have compared outcomes in screen‐detected (asymptomatic) groups with outcomes in elective patients diagnosed after the onset of symptoms,^4^, ^5^ to the best of our knowledge no studies have compared patients specifically in right‐sided colon cancer. There is increasing evidence to support the differing characteristics and disparate outcomes between right and left‐sided cancers, including genomics, complication rates, response to adjuvant chemotherapy, and recurrence rates.^6^, ^7^, ^8^, ^9^, ^10^ Whilst studies have shown increased rates of mortality in emergent versus elective colectomy in symptomatic individuals,^11^, ^12^, ^13^ little is known about how these rates compare specifically in right‐sided colectomy.

A formal study would benefit the consent process and help identify the effect of screen‐detected resections on long‐term post‐resection outcomes. Unlike previous studies, such analyses may also help make inferences about the impact on outcomes at the population level.

The present study aims to report relative outcomes of right‐sided colectomy in screen‐detected colon cancer, compared to elective and emergency symptomatic cases and aims to report the change in outcomes at the population‐level.

## 2 METHODS

### 2.1 Study design

We undertook a prospective observational cohort study of patients undergoing right‐sided colectomy for colon cancer from January 2010 to December 2020 in a tertiary care unit, reported according to the Strengthening the Reporting of Observational Studies in Epidemiology guidelines.^14^ The population of the region was approximately 420,000 people. Data usage approval was granted by our institutional review board and the study was conducted in accordance with the Declaration of Helsinki.

All patients aged over 18 years undergoing right‐sided resection for colorectal cancer were identified from institutional electronic records. Patients where the procedure was not performed for curative intent were excluded (*n* = 92).

### 2.2 Data variables

Background patient characteristics were recorded as follows: demographics, comorbidities, American Society of Anesthesiologists (ASA) score, metabolic equivalents score, smoking history, body mass index, colon cancer symptoms presentation, and referral pathway. The type of right‐sided colonic resection (e.g. right hemicolectomy, extended right hemicolectomy) was performed at the discretion of the surgeon and based on the clinical suspicion and local invasion of the tumor. The surgical approach (laparoscopic vs. open), the need for conversion, the urgency of the procedure and the need for a stoma were documented.

Histopathological data were extracted from postoperative pathology reports and malignant tumors were included. The histological type of cancer (e.g. mucinous adenocarcinoma) was noted, as well as differentiation, lymphovascular invasion, T and N stage, the number of lymph nodes and the number of positive lymph nodes. The resection “R1” status was determined on a cut‐off distance from the tumor to the resection margin of <1 mm. Contrast‐enhanced computed tomography of the chest, abdomen, and pelvis were used to determine the stage of each patient. The use of adjuvant chemotherapy was reported. Pre‐operative albumin and hemoglobin (Hb) was recorded (g/dl).

### 2.3 Outcomes

Patients were grouped by the method of detection of right‐sided colon cancer. Patients were grouped as either screen‐detected or symptomatic groups (including either emergency or elective resections). Emergency groups included those who underwent a resection during an acute admission, whereas the elective group underwent planned resections, often following inpatient or general practitioner investigation. In 2017 fecal occult blood (FOB) was replaced nationally by fecal immunochemical test (FIT) as the screening test for the population. There were no cases of inherited colorectal cancer syndrome in the cohort. Cases of dysplastic polyps under surveillance were included in the elective cohort (*n* = 22).

Clinicopathological variables were reported for each group and were compared between screen‐detected and symptomatic groups. Each patient was then followed up for recurrence and survival data and this was reported using Kaplan–Meier graphs. The length of follow‐up for each patient was recorded and the time to recurrence or death, if applicable, were recorded. Data was censored for patients without recurrence or still alive at the end of follow‐up.

### 2.4 Statistical analysis

R studio version 2022.02.1 was used for the statistical analysis. The Kruskal–Wallis test was applied for the comparison of non‐parametric variables. The chi‐square test was used for the categorical variables and Fisher's exact test when appropriate. The Mann–Kendall trend was performed to assess if there was a trend in the rate of emergency or elective resections over time and the z‐value was reported. A significance level of 0.05 was considered statistically important. Kaplan–Meier survival curves in combination with the logrank test were applied for the estimation of disease‐free survival (DFS), overall survival (OS), and recurrence.

## 3 RESULTS

**FIGURE 1:**
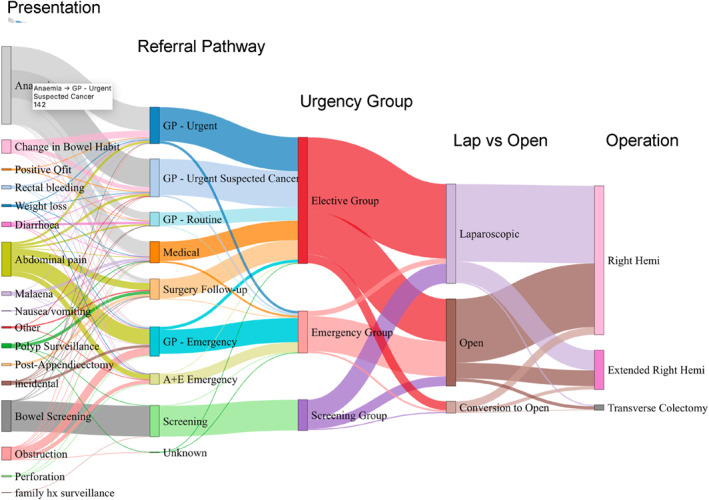
Sankey Diagram—Patient pathway from presentation to operation.

A total of 909 patients (median age, 70; interquartile range [IQR], 58–82; male, 52%) with malignant right‐sided colon cancer undergoing resection with curative intent were included comprising of 151 patients (16.6%) in the screen‐detected group and 758 symptomatic patients (83.4%), including 598 patients undergoing an elective colectomy and 160 patients undergoing emergency colectomy. There was a reducing trend in emergency resections over time (*Z* = −1.89) with borderline statistical significance (*p* = 0.059) but there were no significant trends in elective (*Z* = 0; *p* = 1) or screen‐detected resections (*Z* = 0; *p* = 1). Of the 92 that were excluded for a palliative resection, 5 were screen‐detected (3.2% of screen‐detected overall), 50 were emergency resections (23.8% of emergency resections) and 37 were elective resections (5.8% of elective resections). Figure 1 summaries the presentation, referral pathways, urgency group, and surgical approach of the cohort.

### 3.1 Clinicopathological characteristics

Table 1 illustrates the differences in comorbidities, symptoms, pre‐operative diagnosis, and blood tests prior to resection. Patients presenting under the screen‐detected group were asymptomatic but there were some significant differences in symptoms between emergency and elective group (Table 1).

**TABLE 1:** Preoperative characteristics of emergency, elective and screening right‐sided colectomy.

	Symptomatic patients		Bowel screening, *N* = 151 (%)	*p*‐value (screening vs. emergency)	*p*‐value (screening vs. elective)
	Emergency, *N* = 160 (%)	Elective, *N* = 598 (%)			
Median age (IQR)	71.1	73.1	66.3	<0.001	<0.001
Male	80 (50.0)	274 (45.8)	72 (47.7)	0.683	0.682
BMI (IQR)	27.5	27.6	29	0.011	0.006
Comorbidities					
• Diabetes	26 (16.3)	109 (18.2)	27 (17.9)	0.702	0.921
• Cardiovascular disease	74 (46.3)	338 (56.5)	69 (45.7)	0.922	0.017
• CKD ≥3	20 (12.5)	44 (7.4)	7 (4.6)	0.014	0.235
• Stroke/CNS	11 (6.9)	69 (11.5)	13 (8.6)	0.567	0.303
• COPD	15 (9.4)	73 (12.2)	18 (11.9)	0.466	0.923
• Smoker	24 (15.0)	58 (9.7)	18 (11.9)	0.427	0.419
• Ex smoker	41 (25.6)	61 (10.2)	10 (6.6)	<0.001	0.180
Median METS score	7	7	7	0.192	<0.001
Median ASA score	2	2	2	0.001	<0.001
Main symptom					
• Abdominal pain	66 (41.3)	80 (13.4)	N/A	N/A	N/A
• Anemia	12 (7.5)	364 (60.9)	N/A	N/A	N/A
• Change in bowel habit	4 (2.5)	58 (9.7)	N/A	N/A	N/A
• Diarrhea	6 (3.8)	13 (2.2)	N/A	N/A	N/A
• Incidental	2 (1.3)	25 (4.2)	N/A	N/A	N/A
• Obstruction	41 (25.6)	2 (0.3)	N/A	N/A	N/A
• Post appendicectomy	0 (0.0)	11 (1.8)	N/A	N/A	N/A
• Rectal bleeding	3 (1.9)	12 (2.0)	N/A	N/A	N/A
• Polyp surveillance	0 (0.0)	22 (3.7)	N/A	N/A	N/A
• Other	12 (2.5)	25 (8.4)	N/A	N/A	N/A
Pre‐operative diagnosis					
• Appendix tumor	0 (0.0)	20 (3.3)	0 (0.0)	0.992	0.023
• Caecal cancer	62 (38.8)	184 (30.8)	43 (28.5)	0.056	0.584
• Ascending colon tumor	42 (26.3)	216 (36.1)	46 (30.5)	0.410	0.193
• Proximal transverse colon tumor	21 (13.1)	88 (14.7)	29 (19.20)	0.145	0.175
• Distal transverse colon tumor	18 (11.3)	34 (5.7)	18 (11.9)	0.853	0.007
• Other	14 (8.8)	9 (1.5)	0 (0.0)	<0.001	0.129
Syncronous tumor	2 (1.3)	22 (3.7)	2 (1.3)	0.954	0.142
Any metastases	8 (5.0)	31 (5.2)	6 (4.0)	0.663	0.540
• Peritoneal	2 (1.3)	13 (2.2)	0 (0.0)	0.168	0.068
• Liver	3 (1.9)	16 (2.7)	5 (3.3)	0.424	0.672
• Lung	2 (1.3)	1 (0.2)	0 (0.0)	0.168	0.615
Median Hb	12.3	12	13.2	<0.001	<0.001
Median albumin	29.2	34.7	36.6	0.196	<0.001

Abbreviations: METS, Metabolic Equivalents Score; ASA, American Society of Anaesthesiologists Score; CNS, Central Nervous System; CKD, Chronic Kidney Disease; COPD, Chronic Obstructive Pulmonary Disease; Hb, Haemoglobin.

### 3.2 Operative and histopathological analysis

Operative characteristics were compared between the screen‐detected group and symptomatic groups (Table 2). Significant findings included the higher rate of extended right hemicolectomy (*p* = 0.014) and laparoscopic resection (*p* < 0.001) in the screen‐detected group compared to emergency and elective groups. Rates of ileostomy (both end and loop) were statistically lower in the screen‐detected group versus others (*p* < 0.001 and *p* = 0.043, respectively).

**TABLE 2:** Operative characteristic.

	Symptomatic patients		Bowel screening, *N* = 151 (%)	*p*‐value (screening vs. emergency)	*p*‐value (screening vs. elective)
	Emergency, *N* = 160 (%)	Elective, *N* = 598 (%)			
Type of surgery					
Right hemicolectomy	112 (70.0)	431 (72.1)	100 (66.2)	0.475	0.157
Extended right hemicolectomy	39 (24.4)	106 (17.7)	41 (27.2)	0.575	0.009
Transverse colectomy	3 (1.9)	15 (2.5)	2 (1.3)	0.700	0.382
Right hemi + liver segmentectomy	1 (0.6)	6 (1.0)	2 (1.3)	0.528	0.732
Right hemi, peritonectomy and hyperthermic intraperitoneal chemotherapy	0 (0.0)	15 (2.5)	0 (0.0)	0.997	<0.001
Other	4 (2.5)	15 (2.5)	6 (4.0)	0.462	0.330
Approach					
Laparoscopic	18 (11.3)	365 (61.0)	98 (64.9)	<0.001	0.383
Laparoscopic converted to open	7 (4.4)	35 (5.9)	7 (4.6)	0.912	0.561
Laparotomy	135 (84.4)	196 (32.8)	46 (30.5)	<0.001	0.587
Stoma					
Ileostomy (end)	26 (16.3)	5 (0.8)	1 (0.7)	<0.001	0.830
Ileostomy (loop)	0 (0.0)	12 (2.0)	0 (0.0)	0.997	<0.001
Colostomy	2 (1.3)	2 (0.3)	0 (0.0)	0.168	0.477
No stoma	132 (82.5)	579 (96.8)	149 (98.7)	<0.001	0.218

The comparison of pathological variables is reported in Table 3 where the differences in T stage, nodal status, and R status are reported. Screen‐detected patients had significantly lower stage tumors (*p* < 0.001) and lower rates of lymph node involvement but higher rates of R0 resection (*p* < 0.001).

**TABLE 3:** Pathology results.

	Emergency, *N* = 160 (%)	Elective, *N* = 598 (%)	Bowel screening, *N* = 151 (%)	*p*‐value (screening vs. emergency)	*p*‐value (screening vs. elective)
Biopsy results					
Adenocarcinoma	34 (21.3)	471 (78.8)	118 (78.1)	<0.001	0.869
Adenoma (HG dysplasia)	7 (4.40)	38 (6.4)	22 (14.6)	0.002	0.001
Other	4 (2.5)	17 (2.8)	7 (4.6)	0.308	0.264
Differentiation grade					
Well	2 (1.3)	17 (2.8)	5 (3.3)	0.221	0.761
Moderate	106 (66.3)	418 (69.9)	120 (79.5)	<0.001	<0.001
Poor	41 (25.6)	143 (23.9)	19 (12.6)	0.004	0.003
T‐stage					
T1	1 (0.6)	23 (3.8)	22 (14.6)	<0.001	<0.001
T2	5 (3.1)	53 (8.9)	25 (16.6)	<0.001	0.006
T3	58 (36.3)	366 (61.2)	79 (52.3)	0.004	0.047
T4	83 (51.9)	140 (23.4)	23 (15.2)	<0.001	0.030
N stage					
N0	67 (41.9)	380 (63.5)	104 (68.9)	<0.001	<0.001
N1	44 (27.5)	132 (22.1)	33 (21.9)	0.249	0.954
N2	37 (23.1)	78 (13.0)	12 (7.9)	<0.001	0.085
Average lymph node count	21.41	22	21.6	0.643	0.779
Average negative lymph node count	18.75	20.67	20.72	0.018	0.711
Average positive lymph node count	2.66	1.35	0.95	<0.001	0.208
R0 resection	127 (79.4)	558 (93.3)	148 (98.0)	<0.001	0.026
R1 resection	20 (12.5)	27 (4.5)	1 (0.7)	<0.001	0.026
R2 resection	3 (1.9)	5 (0.8)	0 (0.0)	0.091	0.260

### 3.3 Post‐operative outcomes

Screen‐detected patients had a significantly shorter length of stay than emergency and elective patients, (*p* < 0.001), and no mortality. On the contrary, the emergency group showed higher postoperative mortality rates and were more likely to develop complications (Table 4).

**TABLE 4:** Postoperative outcomes.

	Emergency, *N* = 160 (%)	Elective, *N* = 598 (%)	Bowel screening, *N* = 151 (%)	*p*‐value (screening vs. emergency)	*p*‐value (screening vs. elective)
Inpatient post‐op USS	3 (1.9)	15 (2.5)	1 (0.7)	0.343	0.161
Inpatient post‐op CT	36 (22.5)	123 (20.6)	27 (17.9)	0.311	0.461
1	25 (15.6)	78 (13.0)	19 (12.6)	0.442	0.880
2	4 (2.5)	33 (5.5)	3 (2.0)	0.760	0.070
≥3	7 (4.4)	12 (2.0)	4 (2.6)	<0.001	<0.001
Superficial infection	33 (20.6)	71 (11.9)	19 (12.6)	0.057	0.811
Deep surgical site infection	14 (8.8)	39 (6.5)	8 (5.3)	0.235	0.580
Anastomotic leak	12 (7.5)	25 (4.2)	6 (4.0)	0.183	0.909
Re‐operation	14 (8.8)	28 (4.7)	8 (5.3)	0.235	0.752
I/R intervention	0 (0.0)	16 (2.7)	2 (1.3)	0.144	0.333
Clavien‐Dindo I	15 (9.4)	50 (8.4)	14 (9.3)	0.975	0.721
Clavien‐Dindo II	45 (28.1)	148 (24.7)	38 (25.2)	0.555	0.916
Clavien‐Dindo III	11 (6.9)	28 (4.7)	11 (7.3)	0.888	0.198
Clavien‐Dindo IV	1 (0.6)	3 (0.5)	1 (0.7)	0.967	0.809
Clavien‐Dindo V	11 (6.9)	15 (2.5)	0 (0.0)	0.001	0.049
No complication	77 (48.1)	354 (59.2)	87 (57.6)	0.094	0.724
Post‐op chemo	53 (33.1)	147 (24.6)	54 (35.8)	0.625	0.006
Readmission	16 (10.0)	62 (10.4)	18 (11.9)	0.587	0.581
Recurrence	40 (25.0)	99 (16.6)	13 (8.6)	<0.001	0.014
LOS, days (median)	10	7	6	<0.001	0.011

Abbreviations: LOS, length of stay; USS, Ultrasound.

**FIGURE 2:**
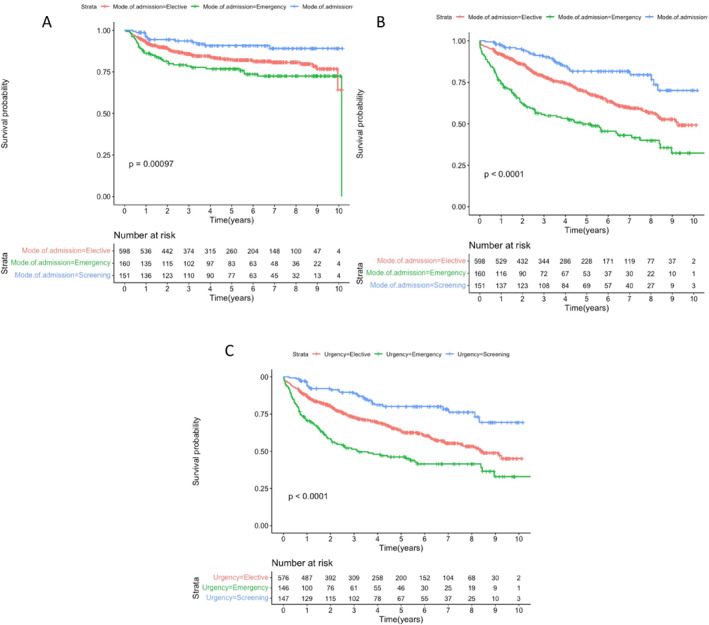
Long term recurrence (A), disease‐free survival (B) and death (C) by urgency of procedure.

In the long term, 25% of the emergency group suffered from disease recurrence, which was significantly higher than the elective (16.6%) and screen‐detected groups (8.6%; *p* < 0.001). Specifically, at 1 year the percentage of patients free from recurrence was estimated at 96% for the screen‐detected group, 92.3% for the elective, and 86.2% for the emergency group; at 3 years, the rates were 94%, 87.1%, and 78.7%, respectively; and at 5 years the rates of recurrence were 92%, 84.9%, and 77.5% respectively. Recurrence for the three groups was illustrated using Kaplan‐Meier (Figure 2).

Patients who had screen‐detected right‐sided colon cancer had higher OS compared to symptomatic patients, as illustrated in the Kaplan–Meier analysis (Figure 2). In particular, 1‐year OS was estimated at 98% for screen‐detected group, 91.9% for elective, and 74.3% for the emergency group; 3‐year OS was 92%, 80.6%, and 57.5%, respectively; and 5‐year OS was 85.4%, 75.4%, and 53.1%, respectively. DFS is also reported for years 1, 3, and 5 (Figure 2).

## 4 DISCUSSION

The present study demonstrates the disparate outcomes between screen‐detected right‐sided colon cancers that proceed to resection in comparison to those that undergo resection in the elective and emergency setting. The results outline that the screen‐detected group are less comorbid overall, have less advanced disease and have a more favorable post‐operative course, including lower rates of complication, recurrence and mortality.

It has been demonstrated that 2‐year OS outcomes are significantly improved for elective versus emergent primary tumor resection in stage IV colorectal cancer, with 80% of elective patients surviving to 2 years versus 42.9% in the emergent cases (*p* = 0.002).^12^ Similar findings are demonstrated once accounting for tumor stage: one study showed that the 5‐year survival for elective cases of tumor resection was 64.4% versus 35.6% (*p* < 0.001) in the emergency cases in groups matched to tumor stage.^13^ It also found that overall DFS is higher in the elective versus emergency cases of resection (75.4% vs. 54.2%, *p* < 0.001).

There is increasing evidence to support the disparate outcomes of right and left hemicolectomy, such as differing complication rates, response to adjuvant chemotherapy, and recurrence rates.^6^, ^7^, ^8^, ^9^, ^10^ Therefore, these two cancers should be treated as separate entities and investigated individually. Genetic analysis has confirmed differing pathways of progression of these pathways through the identification of microsatellite instability and chromosomal instability. Despite recent advancements in immunotherapy, surgical resection is widely considered to be the mainstay of treatment in right‐sided tumors.^15^, ^16^

While previous studies have compared survival between elective and emergency resection, there is little research comparing long term outcomes (e.g. 5‐year DFS) in specifically right‐sided colectomy. More specifically, differences between screen‐detected colon cancers and both elective and emergency groups are largely unknown. One previous study found a statistically significant difference in the OS in screen‐detected patients (5‐year survival rate of 83.7%) versus symptomatic patients (62.3%), but this included right and left‐sided cancer and did not include emergency resections. This study speculated that the effect on survival may be more pronounced in right‐sided colon cancers due to their more aggressive nature and detection difficulties.^4^ A large multicentre Dutch study found a lower postoperative mortality in screen‐detected colon cancers (0.6%) compared to symptomatic patients (1.9%) colon cancers.^5^ Interestingly this paper showed a higher rate of post‐operative mortality in screen‐detected rectal cancer versus symptomatic rectal cancer (Adjusted Odds Ratio, 2.27; 95% confidence intervals [CI], 1.31–3.96), which emphasizes the importance of site‐specific analysis when comparing outcomes relating to bowel‐screening.

The development of novel colorectal cancer screening tools, such as the FOB test (FOBT) and the now‐preferred FIT, have helped to reduce incidence of colorectal cancer by around 6% since 2006.^17^ The current National Bowel Cancer Screening program in Scotland aims to identify individuals in the early, asymptomatic stage of bowel cancer, or with presence of pre‐cancerous polyps.^18^ As demonstrated from this data, in those with an established right‐sided cancer, the screening program may help to identify earlier‐stage cancers in younger patients with less comorbidities compared to symptomatic patients, either elective and emergency.^11^, ^19^

As evidenced by the present data, the screening program appears to have a positive impact on outcome in those patients undergoing resection for right‐sided colon cancer. The screen‐detected group had a lower long‐term recurrence rate, lower rates of postoperative complication, and superior survival. Interestingly, the difference was not limited to screening versus emergency patients; the difference in outcome is also evident between screen‐detected cancers and elective (non‐screen detected) patients. This can be explained by the lower stage of cancer patients but also through the less comorbid and younger screen‐detected cohort.

It is important to acknowledge that the relatively small cohort size in our study compared to the national population may limit the generalizability of these findings to the broader population. Furthermore, the comparison of data between groups is limited to operative candidates and therefore conclusions relating to the true clinicopathological data of all screening patients is not represented by this study. Due to lead‐time bias, the survival benefit in the screen‐detected group may be more accurately explained by earlier detection of the tumor rather than a true survival benefit. Lastly, length‐time bias could also be present whereby more indolent tumors progress slowly without causing symptoms or complication and by their very nature may be associated with the screen‐detected group compared to symptomatic groups, both emergency or elective.

Regardless of the limitations, the results should be regarded as preliminary evidence to guide the consent process and peri‐operative decision‐making based on the method of detection of right‐sided colon cancer and is derived from outcomes of patient groups who proceed to resection. The results are also evidence of the disparate long‐term oncological outcomes between groups based on the method of detection.

## AUTHOR CONTRIBUTIONS

**Thomas Trinder**: Conceptualization; Formal analysis; Resources; Writing ‐ original draft. **Ellie Simeonidou**: Conceptualization; Writing ‐ original draft. **Kate Homyer**: Conceptualization; Data curation; Supervision; Writing ‐ original draft. **Hassan Baig**: Conceptualization; Data curation; Formal analysis; Investigation. **Pradeep Patil**: Conceptualization; Data curation; Methodology; Writing ‐ original draft. **Girivasan Muthukumarasamy**: Conceptualization; Data curation; Methodology; Resources; Writing ‐ review & editing.

## CONFLICT OF INTEREST STATEMENT

The authors declare no conflicts of interest.

## ETHICS STATEMENT

Ethical approval was granted by the regional ethical review board.

## Data Availability

Data can be provided upon reasonable request.
